# Application of green tea catechins, polysaccharides, and flavonol prevent fine dust induced bronchial damage by modulating inflammation and airway cilia

**DOI:** 10.1038/s41598-021-81989-9

**Published:** 2021-01-26

**Authors:** Juewon Kim, Hyunjung Choi, Dong-Hwa Choi, Kyuhee Park, Hyung-June Kim, Miyoung Park

**Affiliations:** 1 R&D Unit, Amorepacific Corporation, Yongin, 17074 Republic of Korea; 2 Gyeonggido Business & Science Accelerator, Suwon, 16229 Republic of Korea

**Keywords:** Chemical biology, Environmental sciences, Natural hazards, Risk factors

## Abstract

Airborne fine dust particles (FDPs) have been identified as major toxins in air pollution that threaten human respiratory health. While searching for an anti-FDP reagent, we found that green tea extract (GTE) and fractions rich in flavonol glycosides (FLGs) and crude tea polysaccharides (CTPs) had protective effects against FDP-stimulated cellular damage in the BEAS-2B airway epithelial cell line. The GTE, FLGs, and CTPs significantly increased viability and lowered oxidative stress levels in FDP-treated cells. Combined treatment with GTE, FLGs, and CTPs also exerted synergistic protective effects on cells and attenuated FDP-induced elevations in inflammatory gene expression. Moreover, the green tea components increased the proportion of ciliated cells and upregulated ciliogenesis in the airway in FDP-stimulated BEAS-2B cells. Our findings provide insights into how natural phytochemicals protect the airway and suggest that green tea could be used to reduce FDP-induced airway damage as an ingredient in pharmaceutical, nutraceutical, and also cosmeceutical products.

## Introduction

Ambient air pollution is composed of gaseous constituents and airborne fine dust particles (FDPs). FDPs can be classified on the basis of the particle size, also known as the aerodynamic equivalent diameter, as smaller than 10 μm, 2.5 μm, or ultrafine size. FDPs reaches the lower airways and accumulates in the more proximal conducting airways^1^. FDPs is naturally inhaled as a particulate suspension in the air and is deposited in the airway as it passes through the respiratory tract, after which it interacts with airway cells. Human bronchial epithelia are invariably exposed to toxic factors such as FDPs, which can cause acute and chronic pulmonary infections and respiratory diseases^2^. In addition, toxic pollutants induce oxidative stress and imbalance between reactive oxygen species (ROS) production and scavenging^3^. Elevated oxidative stress causes airway epithelial barrier dysfunction, airway inflammation, infection, and mitochondrial dysfunction^4^. Airway defense strategies include coughing, anatomical barrier-mediated blockade, immune mechanisms, and primary defense via mucociliary clearance (MCC). MCC enables the efficient clearance of inhaled particles, and in human studies, MCC has been found to remove inhaled particles larger than 6 μm from the airway within 24 h^5^. Because of the toxic effects of FDPs, removal of air pollutants via activation of MCC and attenuation of FDP-induced oxidative stress in airway cells via ROS scavenging are important. Moreover, FDP-generated ROS cause airway cilia dysfunction^6^, and applied ROS scavengers may show positive effects in reducing oxidative stress and activating MCC.

In this study, we focused on the potent activities of natural phytochemicals in green tea. Green tea is an important dietary product that contains antioxidative molecules with cytoprotective and anti-inflammatory activities that protect cells from oxidative stress-induced apoptosis^7^. Catechin compounds, such as (–)-epigallocatechin-3-gallate (EGCG), act as radical scavengers and metal-chelating agents and play roles in various cellular processes; for example, they exert neuroprotective functions, regulate blood pressure, and protect against cardiovascular disease^8^. Previous studies have reported the protective effects of plant extracts and phenolic compounds against oxidative stress and inflammation induced by FDPs^9^, and we hypothesized that green tea components could exert potent protective effects against FDP-induced toxicity. Similar to N-acetyl cysteine, EGCG exhibits scavenging efficacy against FDP-induced ROS^10^. EGCG also reduces skin inflammation and asthma in rats caused by FDP stimulation^10^. However, the studies that have revealed these findings have concentrated only on active EGCG. In addition to catechins, green tea also contains significant amounts of flavonols and polysaccharides^11^. Plant flavonols exhibit anticancer, proapoptotic, antioxidant, antibacterial, and antifibrotic effects^12^. Additionally, polysaccharides exert several health-promoting effects, such as antibacterial, antitumor, antioxidant, and anti-inflammatory effects^13,14^. Despite exerting these positive effects, green tea flavonols and polysaccharides, unlike catechins, have received little attention with respect to their potential biological functionality and possible use.

In the present study, we investigated the protective effects of green tea catechins, flavonols, and polysaccharides against FDP-induced airway cellular toxicity, oxidative stress, and cilia dysfunction using human bronchial epithelial cells (BEAS-2B cells) as an experimental model.

## Results

### FDPs induce oxidative damage and cell death in airway/lung cells

**Figure 1:**
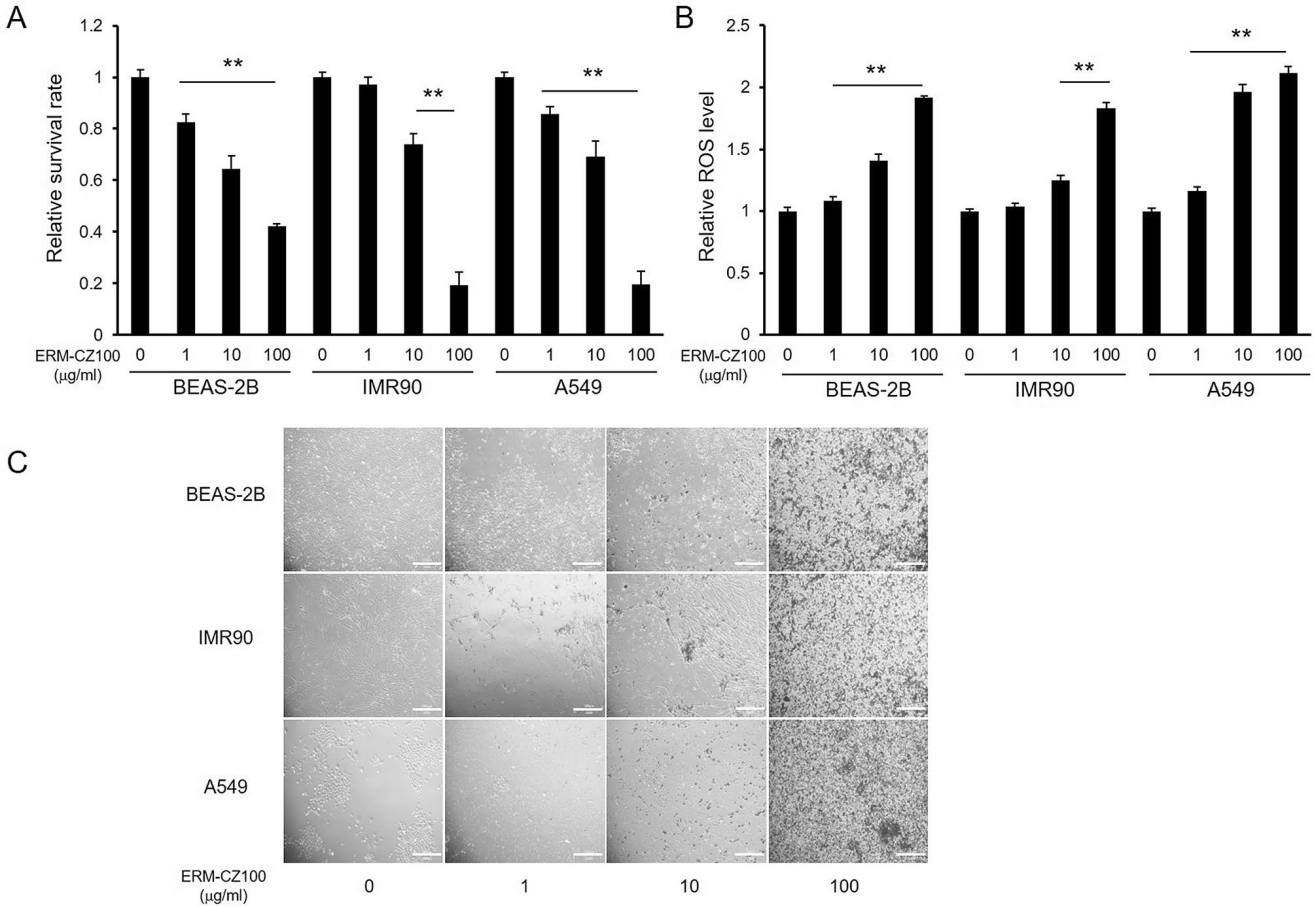
Effects of FDPs on airway and lung cells. (**A**) Cytotoxicity of FDPs toward BEAS-2B, IMR90, and A549 cells. (**B**) Intracellular ROS levels in FDP-stimulated BEAS-2B, IMR90, and A549 cells. (**C**) Cell morphology of FDP-treated cells. Cell viability was estimated by MTT assay, and intracellular ROS levels were determined by DCF-DA assay. The data are presented as the mean ± SD (N = 3). *p < 0.001 compared to the control group. Scale bars, 200 μm.

Following inhalation, the primary sites of air pollution exposure are respiratory tract cells, including bronchial and lung cells. Inhaled FDPs can interact with the epithelial cells lining the airway and with lung cells. Since FDPs are known to cause toxicity in various individual cell types^15^, we attempted to identify the cellular toxicity and oxidative stress levels in airway and lung cells exposed to FDPs. We evaluated cell survival rates and intracellular ROS levels under FDP treatment to examine the cellular damage induced by FDPs. We first performed the experiments using the bronchial epithelial cell line BEAS-2B, the lung fibroblast line IMR90, and the adenocarcinomic alveolar epithelial cell line A549 to assess the toxicity of FDPs on the overall respiratory tract. Although the ROS level is differ by cell line^16^, treatment with FDPs at concentrations in the range of 1–100 μg/ml resulted in significant concentration-dependent reductions in the survival rates of the cells (Fig. 1A) and increases in the cellular ROS levels (Fig. 1B). The morphology of individual cells was also examined (Fig. 1C). In FDPs-treated cells, proliferation of cells greatly suppressed and aggregated with debris of FDPs as shown in figure. This toxicity of FDPs may results in decreased cell viability. The shape of cells also become irregular and percentage of dendritic cells were reduced. The viability of cells treated with 100 μg/ml FDPs was lower than 50%, and the ROS levels in these cells were nearly twofold those in the control cells. As the damaging effects of FDPs on viability and ROS levels were similar among the tested respiratory tract cell lines, we proceeded to perform further experiments using BEAS-2B bronchial cells, the first airway cells exposed to air pollution.

### Protective effects of the green tea components against FDP-induced damage

**Figure 2:**
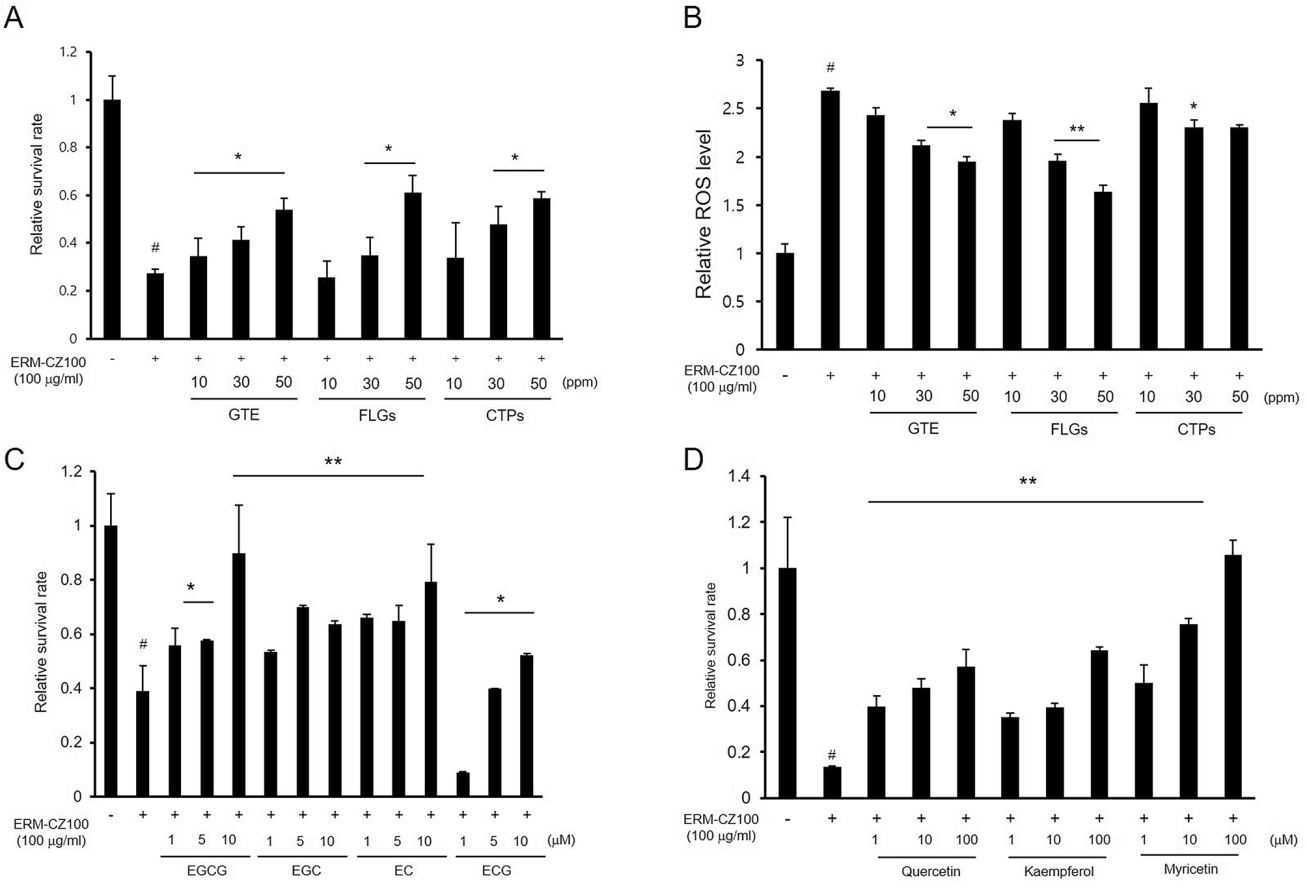
Protective effects of GTE, FLGs, and CTPs and their bioactive components against FDP-induced damage. (**A**) Protective effects of GTE, FLGs, and CTPs against FDP-induced cell death. (**B**) Relative ROS levels in FDP-stimulated cells treated with GTE, FLGs, and CTPs. (**C**) Protective effects of green tea catechins against FDP-induced cytotoxicity. (**D**) Effects of green tea flavonols on the relative survival rates of FDP-treated cells. Cell viability was measured by MTT assay, and intracellular ROS levels were investigated by DCF-DA assay. All results are expressed as the means ± SDs of the values obtained in 3 independent experiments (N = 3). *p < 0.05 and **p < 0.001 compared to the FDP-treated group; ^#^*p* < 0.001 compared to the vehicle-control group.

Experimental studies have shown that extracts and phenolic compounds derived from green tea have antioxidant and anti-inflammatory effects on FDPs-exposed skin cells^10^. However, although green tea contains various bioactive ingredients, the previous studies have focused only on EGCG, a representative catechin component. In addition to EGCG, green tea contains high amounts of bioactive polyphenols, such as flavonols, as well as polysaccharides^17^. Recently, we isolated GTE, fractions rich in FLGs, and CTPs from green tea leaves as bioactive ingredients^18^. Because phenolic compounds from various plants have been shown to exert protective effects against FDP-induced oxidative stress and inflammation in skin cells^19^ and because green tea polysaccharides exhibit beneficial antioxidant^20^, antitumor^13^, and antiaging properties^21^, we hypothesized that green tea polyphenols and polysaccharides could also ameliorate FDP-induced cellular damage in the airway. Our results revealed that GTE, FLGs, and CTPs attenuated the cellular toxicity induced by FDPs in BEAS-2B cells in a concentration range of 10–50 ppm (Fig. 2A). These bioactive green tea ingredients also lowered oxidative stress levels in FDP-treated cells (Fig. 2B).

The chemical properties of GTE, FLGs, and CTPs were investigated. As shown in Table S1, the total catechins in GTE amounted to 36.52 ± 1.6% of the dry matter and included EGCG (16.8 ± 0.8%), EGC (11.8 ± 2.0%), EC (3.68 ± 0.8%), and ECG (2.94 ± 0.2%). On the other hand, catechins, a major group of phenolics in green tea, were not detected, and the FLGs quercetin, kaempferol, and myricetin were contained mainly in the FLG fraction^22^. In addition, the CTPs included mainly pectic substances and glucosidic macromolecules found in unlignified cell walls, major components of the middle lamellae in plants^18^; these pectic polysaccharides have various pharmacological properties^11^. We next examined the effects of higher levels of GTE and FLGs on FDP-induced cellular toxicity in BEAS-2B cells. The epicatechins EGCG, EGC, EC, and ECG effectively reduced FDP-induced cytotoxicity when applied at concentrations ranging from 1 to 10 μM (Fig. 2C). The flavonols quercetin, kaempferol, and myricetin also showed protective effects on cell viability in the 1–100 μM concentration range (Fig. 2D). Among these active compounds, EGCG and myricetin, which were found in the GTE and FLGs, respectively, showed the most potent protective efficacy against FDP.

### Combined effects of the green tea components on FDP-induced cellular damage

According to the cell survival rates under FDP treatment, the catechin EGCG and the flavonol myricetin exhibited potent and concentration-dependent protective effects against FDP-induced toxicity (Fig. 2C,D). EGCG was the most abundant catechin in GTE and is commonly used in cosmetics, functional foods, and dietary supplements due to its health benefits^23^. EGCG has also been shown to reduce FDP-induced skin inflammation in epidermal keratinocytes and dermal fibroblasts^10^. Myricetin is a flavonol that is present in vegetables, fruits, nuts, berries, and tea. Like many other flavonols, myricetin shows antioxidant, antiviral, and anti-inflammatory effects^12^. Interestingly, among polyphenolic compounds, myricetin and EGCG have been shown to exhibit inhibitory effects against house dust-induced allergic reactions^24^. According to a previous report, among polyphenols (including catechins and flavonols), myricetin and EGCG effectively inhibit the release of kinin by house dust mites.

**Figure 3:**
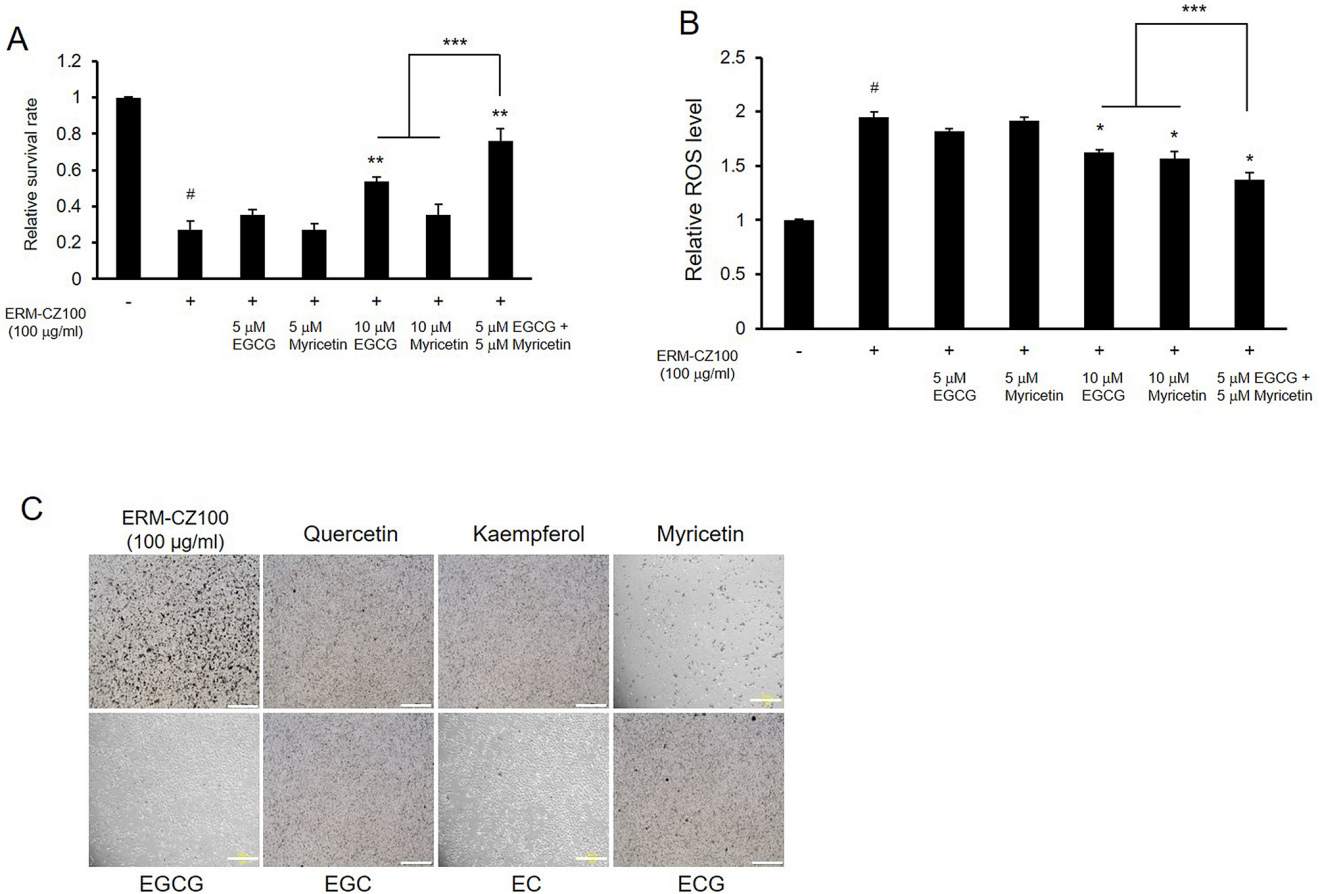
Synergistic effects of EGCG and myricetin against FDP-induced cellular damage. (**A**) Combined protective effects of EGCG and myricetin on cell survival under FDP treatment. (**B**) Synergistic ROS-scavenging effects of EGCG and myricetin in FDP-stimulated BEAS-2B cells. (**C**) Effects of green tea catechins and flavonols on the morphology of FDP-treated cells. The results are shown as the means ± SDs of the values obtained in 3 independent experiments (N = 3). *p < 0.05 and **p < 0.001 compared to the FDP-treated group; ***p < 0.01 compared to the 10 μM alone treatment vs. 5 μM combined treatment; ^#^*p* < 0.001 compared to the vehicle control group. Scale bars, 200 μm.

**Figure 4:**
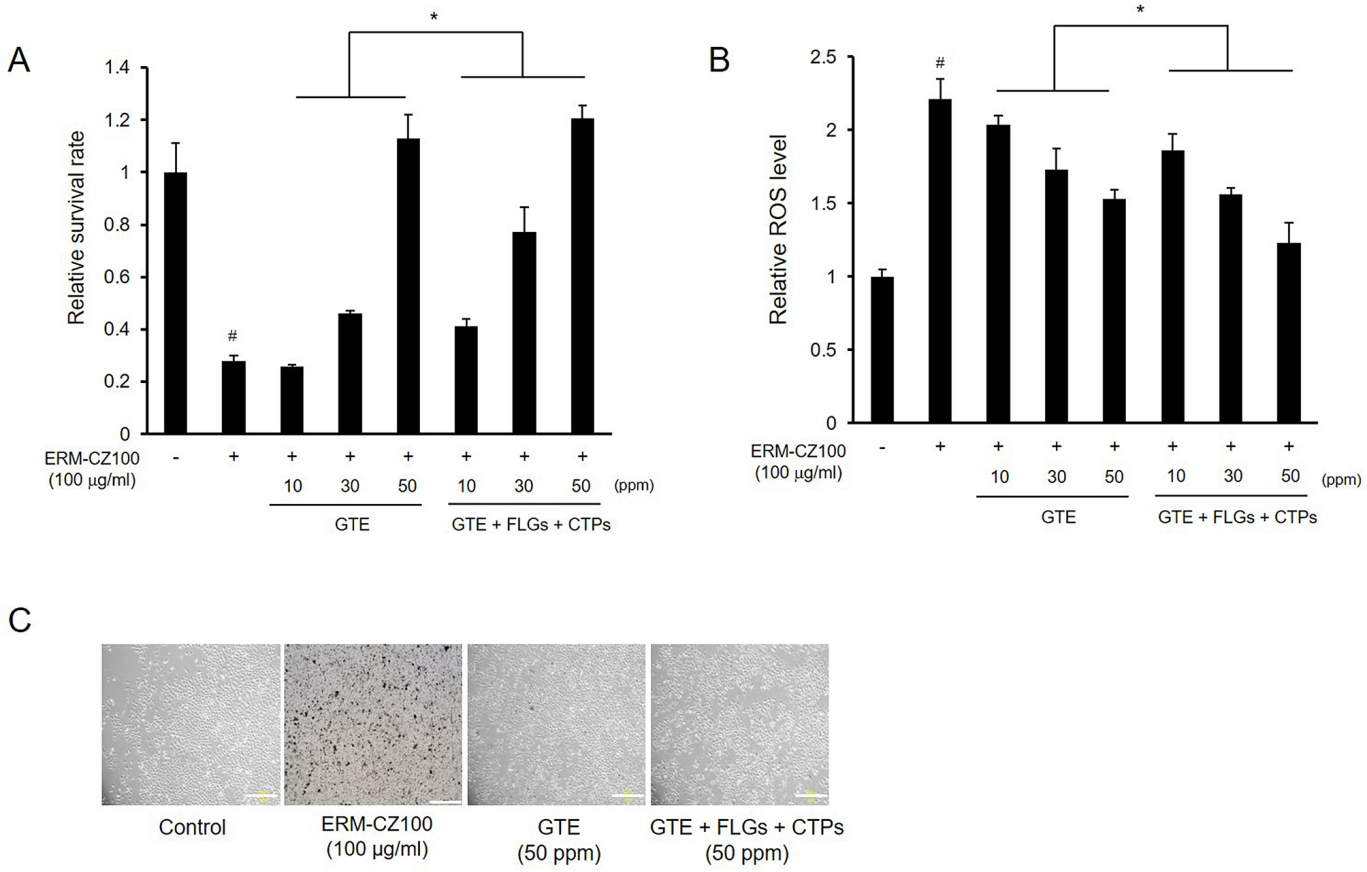
Protective effects of GTE, FLGs, and CTPs against FDP-elicited cellular damage. (**A**) Protective effects of GTE, FLGs, and CTPs on cell survival in FDP-stimulated BEAS-2B cells. (**B**) Protective ROS-scavenging effects of GTE, FLGs, and CTPs against FDP-induced oxidative stress. (**C**) Effects of GTE, FLGs, and CTPs on the morphology of FDP-treated BEAS-2B cells. The data are presented as the mean ± SD (N = 6). *p < 0.05 compared to the component alone treatment with combined treatment; ^#^*p* < 0.001 compared to the vehicle control group. Scale bars, 200 μm.

We tested the effects of EGCG and myricetin on cellular toxicity and ROS levels to investigate whether these active compounds in green tea have synergistic effects. Administration of 10 μM EGCG or myricetin reduced toxicity and intracellular ROS levels in cells under FDP treatment (Fig. 3A,B). Surprisingly, cotreatment with 5 μM EGCG and myricetin exhibited synergistic effects, enhancing cell viability and attenuating oxidative stress levels in FDP-treated cells (Fig. 3A,B). The effects of representative catechins and flavonols on FDP-induced morphological changes are depicted in Fig. 3C. As previously shown, the shape of cell was irregular, cells were aggregated with FDPs, and rate of dendritic cells were decreased. We next tested the combined preventative effects of GTE and the FLG and CTP green tea fractions against FDP-induced cellular damage. Combined treatment with the green tea components was more effective than GTE treatment alone in preventing FDP-induced cell survival impairment and oxidative stress (Fig. 4A–C). Taken together, these data suggest that supplementation with whole green tea rather than with a specific fraction may enable useful to combat FDP-induced cellular damage.

### Protective effects of the green tea components against the FDP-induced immune response

**Figure 5:**
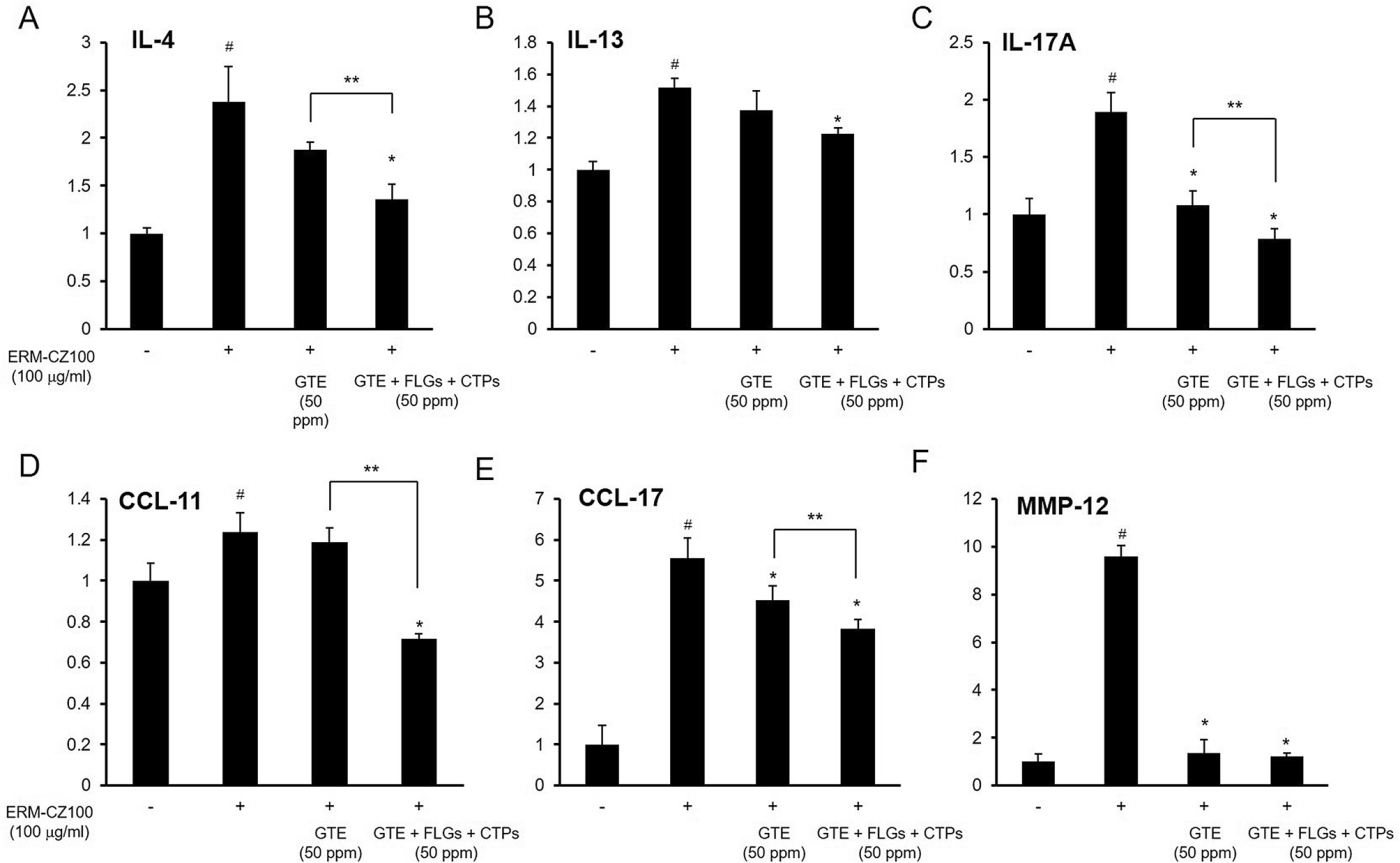
Inhibitory effects of GTE or GTE, FLGs, and CTPs against FDP-induced inflammatory response elevation. The synergistic effects of GTE, FLGs, and CTPs on the expression levels of the airway inflammatory genes (**A**) IL-4, (**B**) IL-13, (**C**) IL-17A, (**D**) CCL-11, (**E**) CCL-17, and (**F**) MMP12 were measured in FDP-stimulated BEAS-2B cells. All results are either representative results or are expressed as the means ± SDs of the values obtained in three independent experiments (N = 3). *p < 0.05 compared to the FDP-treated group and **p < 0.05 compared to combined treatment with GTE; ^#^*p* < 0.001 compared to the vehicle control group.

Following inhalation, the primary site of exposure to FDPs is the airway tract. Inhaled FDPs directly affects the immune processes of airway epithelial cells, which can be stimulated by airborne materials in the environment. FDP-stimulated airway cells act as components of multicellular immune responses and trigger cellular signaling pathways. Because improper and excessive immune reactions can result in serious infections, malignancies, and autoimmune conditions, proper regulation of the effects of air pollution on the immune system is important^15^. The proinflammatory cytokine milieu in the airway that develops after inhalation of FDPs disrupts immune modulation. To determine whether green tea and the combination of GTE, FLGs, and CTPs attenuated FDP-induced effects on the airway immune system, we investigated the expression of known bronchial inflammatory genes. Upon sensing of toxic particles, bronchial epithelial cells produce many pro-inflammatory cytokines^25^. These molecules are well known asthma and chronic obstructive pulmonary disease (COPD) markers^26^. Treatment with 100 μg/ml FDPs greatly increased the expression of the inflammatory marker genes IL-4, IL-13, IL-17A, CCL-11, CCL-17, and MMP-12. Polyphenols modulate inflammatory response by regulating pro-inflammatory cytokines synthesis and gene regulation^27^, we expected the lowering effects of hyper-immune response by green tea component treatment. Combined treatment with GTE, FLGs, and CTPs largely reduced this hyper-immune response to a greater extent than treatment with GTE alone except IL-13 and MMP-12 (Fig. 5). This pollution-induced cytokine response is clinically relevant^28^, and as IL-17A is also known as a COPD marker, the green tea components exhibit potential for the prevention of airway disease. Moreover, given that IL-4, IL-13, CCL-11, and CCL-17 regulate lung cell tight junctions, these results suggest that the green tea components can be used for lung health applications.

### Protective effects of the green tea components on airway cilia

Bronchial epithelial cells create a physical barrier at the airway lumen, form the mucous lining of the respiratory tract, sense toxic biological and anthropogenic FDPs accumulation on the airway wall and help to remove FDPs from the airway via ciliary action. MCC plays a crucial role in the airway defense machinery, as it involves secretion of antimicrobials, fluids, and anti-inflammatory proteins^29^. The mucociliary system removes FDPs and pathogens mechanically via the actions of cilia and coughing^30^. MCC abnormalities related to ciliary dysfunction can result in chronic pulmonary disorders, including asthma and COPD. In patients with primary ciliary dyskinesia, airway clearance of particles is impaired and prolonged, which allows longer residence times for bacteria, viruses, and toxins in the airway^31^. Disruption of ciliated cell functions has been detected in many chronic lung diseases and contributes to morbidity, mortality, and infection in individuals with these disorders^32^.

**Figure 6:**
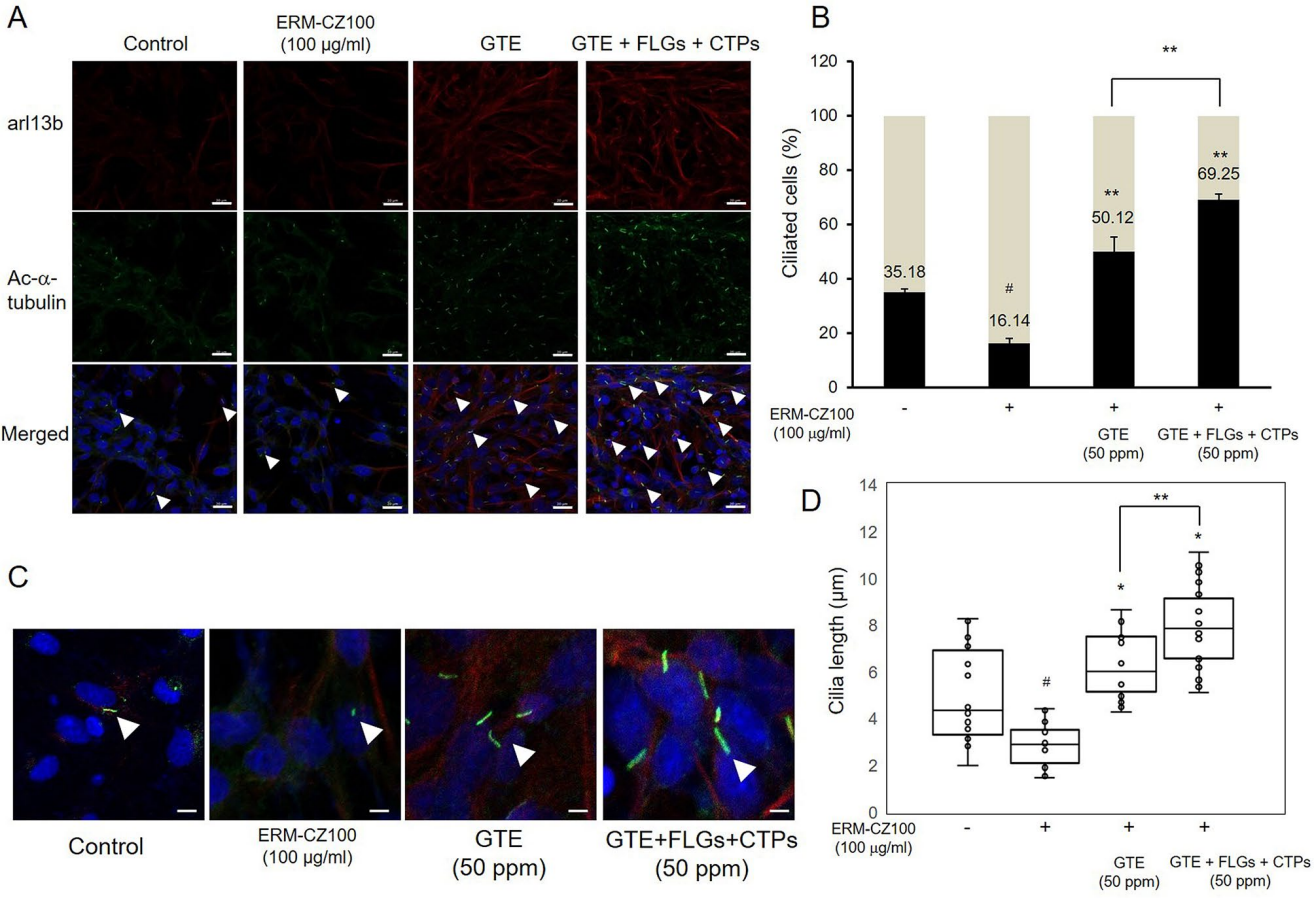
Ameliorative effects of GTE, FLGs, and CTPs on FDP-induced ciliogenesis suppression in BEAS-2B cells. (**A**) Immunostaining of cilia in FDP-stimulated BEAS-2B cells. (**B**) Ciliated cells were counted, and the results are presented as the proportions of ciliated cells among all cells. (**C**) Activation of ciliogenesis by GTE, FLG, and CTP treatment. (**D**) Cilia length of ciliated cells. Boxplot the median with upper and lower quartiles, all data points have been plotted. Data have a minimum of 17 cilia per condition, from a total of between 3 and 6 experiments. The data are presented as the mean ± SD (N = 3). *p < 0.05 compared to the FDP-treated group; **p < 0.001 compared to combined treatment with GTE; ^#^*p* < 0.001 compared to the vehicle control group. The ciliated cells were immunostained for ARL13B (red) and acetylated-α-tubulin (green), and merged fluorescence is indicated with DAPI (blue). Scale bars, 20 μm in A and 5 μm in (**C**).

To determine the effects of the green tea components on ciliogenesis in bronchial epithelial cells, we treated FDP-treated BEAS-2B cells with GTE or with GTE, FLGs, and CTPs. FDP treatment greatly suppressed cilium formation (Fig. 6A,B) and cilium length (Fig. 6C,D). Cilia are specialized microtubule-based cellular organelles that beat in metachronal waves to facilitate the expulsion of inhaled particles and pathogens trapped in the mucus layer from the airway^33^. To detect cilia, immunofluorescence was performed for acetylated α-tubulin and the ARL13B protein^34^. Ciliated cells are defined by their multiple motile apical cilia and by the presence of motor proteins that mediate directional beating, which is critical for MCC^35^. Although the biomechanical actions of cilia in epithelial cells are well identified as essential building blocks of MCC^31^, recent studies have revealed that airway ciliated cells sense and respond to mechanical and irritant stimulation^36^. In our study, we found that GTE increased the proportion of ciliated cells by approximately threefold under FDP treatment conditions; moreover, the combined green tea components increased the ciliated cell proportion by over fourfold (Fig. 6A,B). Additionally, GTE, FLG, and CTP application protected cilia against FDP-induced declines in length (Fig. 6C,D), indicating that these green tea components activate airway cilia and can upregulate MCC of inhaled pollutants such as FDPs.

## Discussion

We depicted here the protective effects of green tea catechins, polysaccharides, and flavonols against airborne particles. Compared to green tea catechins, flavonols and polysaccharides from green tea have received little attention. Green tea contains a many of polyphenols, especially monomeric flavonols. Since there are very limited studies for investigating protective effects of green tea polyphenols against particulate pollutants^37^, we examined green tea flavonols rich fraction FLGs and its representative compounds, quercetin, kaempferol, and myricetin for damage attenuation by FDP. Among these compounds, myricetin have shown potent positive effects of cell survival under FDP-stimulation dose-dependent manner (Fig. 2D). Moreover, myricetin represented synergetic effects against FDP with EGCG. Combination of myricetin with EGCG effectively lowered cellular ROS level and increased cell survival under FDP treatment (Fig. 3A,B). The chemical properties and sugars of CTP fractions compared with GTE, the protein contents were nearly the same^18^. The rhamnogalacturonan-II polysaccharide was enriched in the CTP fraction rather than in GTE, which is known as a unique component of several plant polysaccharides and CTPs also contained low molecular weight (MW) catechins with the addition of high MW polysaccharides. These peculiar components of CTPs may result in synergetic protective effects against FDPs with GTE and FLGs.

Along with green tea catechins and flavonols, polysaccharides are also gaining attention due to their health benefits, especially immune response^38^. Air pollution has increased concern about its inhalation toxicity. Inadequate or excessive immune reactions by FDP could result in serious infection, metastatic malignancies, and auto-immune state. FDP stimulates cells through ROS sensing pathways and activate pro-inflammatory signaling cascades such as MAPK pathways^39^. The affection of FDP to stimulate cells may due to the particles containing microbial molecules and also pollutants inducing host-derived molecules production. FDP could stimulate airway epithelial cells and generate ROS in cellular and acellular systems^40^. FDP also cause oxidative stress by both the heavy metal and organic compounds and can directly reduce endogenous antioxidants^41^. In our results, combination of green tea components effectively inhibited hyper-immune responses by FDP-stimulation (Fig. 5). These pro-inflammatory cytokine milieus after ambient pollutant inhalation may be important for perturbing immune regulation and actually closely related with airway and pulmonary dysfunction, such as asthma and COPD. Treatment of whole green tea component could be more promising application to attenuate immune dysfunction more than green tea catechin only. Although in vitro experiments are important for understanding the toxicity of air pollution and its therapeutic treatment, pollution exposure in cell culture system has limitation of actual inhalation. However, relationship between airway defenses and epithelial cells gives a clue to protect airway against FDP.

FDP in naturally inhaled as a particulate matter in the air, deposit in the airway, passing through respiratory tract before interacting with airway cells. The bronchial airway cells form a physical barrier, sense dangerous biological and toxic particles, and deposit on the airway wall by ciliary action to clear particles from the airway. The airway cilia are an organelle producing from the cell body that senses external stimulations and removes harmful particles by its movements. Mucociliary clearance is an integral part of airway-lung defense methods, enabling efficient clearance of inhaled particles from the respiratory tract^2^. The activity and efficacy of clearance closely related with ciliated cells proportion and ciliogenesis in airway epithelial cells^33^. As shown in our results, application of green tea component enhanced the ciliated cells rate of BEAS-2B cell and also showed increased cilia length against FDP treatment. These ciliogenesis activation was more remarkable in combined treatment of green tea component and it could be upregulate MCC actions against pollutant inhalation.

In this study, we investigated FDP-induced cellular toxicity and oxidative stress in bronchial epithelial cells and examined the protective effects of green tea components against FDP-induced cytotoxicity and declines in ciliogenesis. Our results demonstrated that FDPs induced cytotoxicity by increasing intracellular oxidative stress levels, reducing cell viability, increasing inflammatory gene expression, and attenuating airway ciliogenesis in BEAS-2B cells. Green tea components including catechins, flavonols, and polysaccharides exerted protective effects individually and in combination against FDP-induced cellular damage. Specifically, GTE, FLGs, and CTPs lowered intracellular ROS levels, may result in increased oxidative stress resistance, attenuated the hyperimmune response and increased ciliated cell beating rates and ciliogenesis. Amelioration of acute oxidative stress, proper modulation of the immune response and activation of MCC are critical strategies for protection against air pollution-induced airway/lung damage. Moreover, in this study, we pre-treated green tea components in bronchial cells prior to FDP stimulation. As this regard, we want to emphasize protection effects of green tea component against FDPs with elevated oxidative stress resistance of cells. Based on these results, we suggest that green tea catechins, flavonols, and polysaccharides are promising reagents for protection against FDP-induced airway damage and are candidates for use in the pharmaceutical, nutraceutical, and also cosmeceutical fields.

## Methods

### Chemicals and reagents

The airborne FDPs reference material ERM-CZ100 (smaller than 10 μm) and the pure catechin compounds EGCG, epigallocatechin (EGC), epicatechin (EC), and epicatechin gallate (ECG) were purchased from Sigma-Aldrich (St. Louis, MO, USA). Phenolic compounds, quercetin, kaempferol, and myricetin were also obtained from Sigma-Aldrich. Dried green tea leaves (Osulloc Farm, Jeju Island, Korea) were obtained, extracted, and purified for the preparation of GTE, FLGs, and CTPs. All other chemicals used in this study were of analytical grade.

ERM-CZ100 was suspended in serum-free DMEM and homogenized by sonication to make a 10 mg/ml stock solution. Phosphate-buffered saline (PBS), dimethyl sulfoxide (DMSO), and the fluorescent probe 2′,7′-dichlorodihydrofluorescein diacetate (DCFH-DA) were purchased from Sigma-Aldrich. MitoSOX Red (M36008) was obtained from Thermo Fisher Scientific (Indianapolis, IN, USA), and an MTT assay kit and a Cell Counting Kit-8 were purchased from Dojindo (Kumamoto, Japan).

### Preparation of GTE, purified flavonol glycosides (FLGs), and CTPs from green tea leaves

Dried green tea leaves (Osulloc Farm, Jeju Island, Korea) were obtained, extracted, and purified for the preparation of GTE, FLGs, and CTPs as previously described. Briefly, dried green tea leaves were soaked in 70% (v/v) aqueous ethanol at 70 °C for 1 h. The ethanol in the extract was removed by an evaporator (Hei-VAP, Heidolph Instruments, Schwabach, Germany), and the remaining material was filtered using a 20 μm filter (Pall Corp., Port Washington, NY, USA) and solidified with a KL-8 spray dryer (Seogang Engineering, Cheonan, Korea) to obtain the GTE. To obtain purified FLGs, GTE aqueous solution (1% w/v, pH 5.0) was reacted with 1% (v/v) tannase (500 units/ml) in a thermoshaker (Eppendorf, Hamburg, Germany) for 14 h at 40 °C. The enzymatic reaction was stopped by heating at 90 °C for 20 min. The remaining residues after GTE extraction were extracted with water at 90 °C for 3 h and filtered to remove insoluble residue. The clear supernatant was concentrated to 1:150 (v/v) in a vacuum evaporator and precipitated by supplementation with 4 volumes of 95% cold ethanol to obtain crude polysaccharides. Then, the precipitates were dissolved in a small amount of water and spray-dried to produce the CTP fraction. We treated these fractions as mixture of GTE, FLG, and CTP by mixing same degree of fractions (10, 30, 50 ppm) or individually to the bronchial cells.

### Cell culture

The human bronchial epithelial cell line BEAS-2B (CRL-9609), the human fibroblast cell line IMR90 (CCL-186), and the human lung adenocarcinoma cell line A549 (CCL-185) were purchased from the American Type Culture Collection (ATCC, Manassas, VA, USA). The cell lines were cultured at 37 °C in a humidified atmosphere with 5% CO_2_.

### Determination of the effects of the reagents on cell survival after exposure to fine dust

Cells were incubated in a 12-well plate for 24 h and then treated with different reagents. After 2 days of treatment, FDPs (100 μg/ml) were added to the cells. After 24 h of incubation, MTT solution (2 mg/mL) was added to each well, and the cells were further incubated for 1 h. The cell viability was determined by MTT assay^42^ with a microplate reader (SPECTROstar Nano, BMG Labtech, Ortenberg, Germany).

### Determination of intracellular ROS levels

Cells were seeded in 24-well plates for 24 h and then treated with various reagents. After 2 days of incubation, FDPs (100 μg/ml) were added to the cells. After 6 h, the intracellular ROS levels were measured by 2′,7′-dichlorofluorescein diacetate (DCF-DA) assay (excitation: 485 nm; emission: 535 nm) with a microplate reader (SPECTROstar Nano, BMG Labtech, Ortenberg, Germany).

### RNA extraction and quantitative real-time PCR

Total RNA from BEAS-2B cells was prepared with an RNeasy Mini Kit (#74106, Qiagen, Hilden, Germany). Reverse transcription was performed on 4 μg of total RNA using an iScript cDNA Synthesis Kit (#170-8891, Bio-Rad, CA, USA). Quantitative PCR was performed using an ABI 7500 Fast Real-Time PCR System with TaqMan Universal Master Mix II and TaqMan site-specific primers and probes (Applied Biosystems, CA, USA). All reactions were performed in triplicate, and the amounts of mRNA were calculated by the comparative cycle threshold (CT) method.

### Immunofluorescence labeling of cilia

BEAS-2B cells were treated with each reagent, washed with PBS, fixed for 30 min in 4% paraformaldehyde, washed again, and incubated for 10 min in 0.1% Triton X-100. The cells were washed three times in PBS and incubated with anti-acetylated tubulin antibodies (1:1000 dilution, Sigma-Aldrich, MO, USA) and anti-ARL13B antibodies (1:200 dilution, Proteintech, IL, USA) diluted in Hank’s solution (0.44 mM KH_2_PO_4_, 5.37 mM KCl, 0.34 mM Na_2_HPO_4_, 136.89 mM NaCl, and 5.55 mM d-glucose) at 4 °C overnight. The cells were then incubated with goat anti-rabbit or goat anti-mouse Alexa Fluor 555- or Alexa Fluor 488-conjugated secondary antibodies for 1 h at room temperature. After washing, the coverslips were mounted onto glass slides and visualized using a confocal laser scanning microscope (LSM800, Carl Zeiss, Germany). DAPI was used to counterstain the cell nuclei. The acquired images were analyzed using ZEN software (Carl Zeiss).

### Statistical analysis

The data are expressed as the mean ± SD. The normality of the data was analyzed using the Shapiro–Wilk test, and the results between different groups were compared using one-way ANOVA (followed by Dunnett’s post hoc test) or Student’s t-test. All statistical tests were two-sided, with the level of significance established at *p* < 0.05. SPSS software (ver. 21, SAS Institute, NC, USA) was used for statistical analyses.

## Supplementary Information

Supplementary Information.
