# Supplementary material for: Application of green tea catechins, polysaccharides, and flavonol prevent fine dust induced bronchial damage by modulating inflammation and airway cilia
Source: Sci Rep. 2021 Jan 26;11:2232. doi: 10.1038/s41598-021-81989-9 (PMC7838266; doi:10.1038/s41598-021-81989-9)
Supplement: Supplementary file 1 — Supplementary Information. [file 41598_2021_81989_MOESM1_ESM.docx]

**Supporting Information**

**Application of green tea catechins, polysaccharides, and flavonol prevent fine dust induced bronchial damage by modulating inflammation and airway cilia**

Juewon Kim, Hyunjung Choi, Dong-Hwa Choi, Kyuhee Park, Hyung-June Kim, Miyoung Park

Table S1

Figure S1-2

Table S1. Chemical properties and identification of catechins in green tea extract (GTE) prepared from the leaves of green tea

| Chemical constituent | Amount in GTE (w/w) (%) |
| --- | --- |
| Epigallocatechin | 11.8 ± 2.0 |
| Gallocatechin | 0.56 ± 0.26 |
| Epicatechin | 3.68 ± 0.8 |
| Catechin | 0.21 ± 0.06 |
| Epigallocatechin gallate | 16.8 ± 0.8 |
| Gallocatechin gallate | 0.53 ± 0.4 |
| Epicatechin gallate | 2.94 ± 0.2 |
| Total catechin | 36.52 ± 1.6 |
| Caffeine | 3.3 ± 0.5 |

Percentage (%) in dry matter. The data are presented as the mean ± SE (N = 5).

Fig. S1. Cell viability of BEAS-2B under treatment with GTE, FLGs, or CTPs for 6-96 hrs. The data are presented as the mean ± SD. (N = 3).


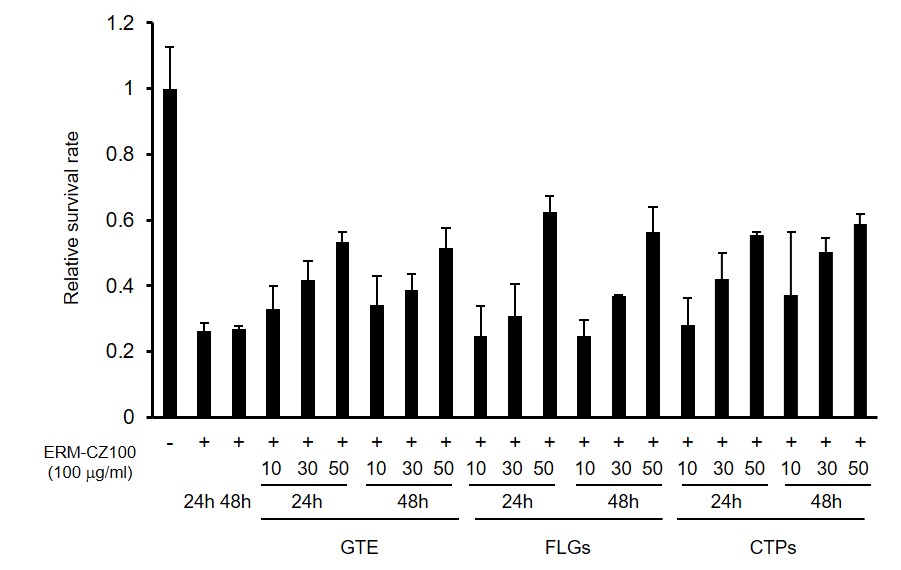


Fig. S2. Cell survival of BEAS-2B with GTE, FLGs, or CTPs under treatment of 100 μg/ml ERM-CZ100 for 24 or 48 hrs. The data are presented as the mean ± SD. (N = 3).
